# Optimizing Surgical Care Amidst COVID-19: A Scoping Review of Practices and Policies

**DOI:** 10.3390/healthcare12010096

**Published:** 2023-12-31

**Authors:** Nasser A. N. Alzerwi, Musaed Rayzah, Ahmad K. Alnemare, Ahmed M. E. Elkhalifa

**Affiliations:** 1 Department of Surgery, College of Medicine, Majmaah University, Ministry of Education, Al-Majmaah City 11952, P.O. Box 66 Riyadh, Saudi Arabia; 2 Otolaryngology Department, College of Medicine, Majmaah University, Ministry of Education, Al-Majmaah City 11952, P.O. Box 66 Riyadh, Saudi Arabia; a.alnemare@mu.edu.sa; 3 Department of Public Health, College of Health Sciences, Saudi Electronic University, Riyadh 11673, Saudi Arabia; a.alkhalifa@seu.edu.sa; 4 Department of Haematology, Faculty of Medical Laboratory Sciences, University of El Imam El Mahdi, Kosti 1158, Sudan

**Keywords:** hospital resources, pandemic, surgery, surgical services, systematic review

## Abstract

Background: The coronavirus disease (COVID-19) pandemic significantly disrupted surgical care worldwide, affecting different specialties in various ways. Lockdowns, surges in COVID-19 cases, and changes in hospital policies notably impacted patient attendance, management practices, and access to surgical services. This scoping review examines the adverse impacts of the COVID-19 pandemic on surgical services and the policies adopted to address these care barriers. Methods: We conducted a comprehensive literature review using the preferred reporting items for systematic reviews and meta-analyses extension for scoping reviews (PRISMA-ScR) guidelines. Our search, spanning 31 December 2019, to 29 January 2023, focused on understanding the multifaceted impacts of COVID-19 on surgical services, particularly across different specialties. Results: An analysis of 75 articles indicated that the pandemic challenged surgeons worldwide to maintain a balance between delivering emergency and elective surgical care, and implementing safety measures against viral transmission. There was a marked decline in the surgical volume, leading to extended waitlists and decreased operating theater usage. Strategies such as prioritizing medically necessary and time-sensitive surgeries and integrating telemedicine have emerged as pivotal for ensuring the continuity of urgent care. Despite the reduced rates, essential surgeries such as appendectomies and cancer-related operations continued, yet faced hurdles, including reduced staffing, limited operating theater capacity, and complications in patient transfers. Conclusions: This review emphasizes the steep reduction in surgical service utilization at the beginning of the pandemic and emergence of new compounded barriers. Policies that designated surgeries as essential, and focused on equitable and timely access, were effective. Incorporating these findings into post-pandemic assessments and future planning is crucial to sustain adequate surgical care during similar health emergencies.

## 1. Introduction

The coronavirus disease 2019 (COVID-19) pandemic, which emerged in late 2019, has profoundly impacted surgical care delivery globally [1]. By February 2023, the World Health Organization reported more than 750 million cases and 6.8 million deaths worldwide [2]. As COVID-19 spread, even hospitals providing complex tertiary procedures became sites of care for infected patients [3,4]. This public health emergency requires major changes to maintain safety and serve surgical patients.

Several key aspects illuminate the influence of pandemics on surgical services. Elective operations experience extensive delays or cancellations to conserve resources and intraoperatively mitigate viral transmission [1]. However, denial of surgical care has negative ramifications, including prolonged pain and disability. Hospitals instituted more stringent protocols, including enhanced disinfection, personal protective equipment, and preoperative screening [5]. Although crucial for infection control, these additional measures constrain surgical volumes. In addition, telemedicine virtual platforms have been rapidly adopted to facilitate remote consultation and follow-up [6]. However, telemedicine cannot completely replace in-person interaction and evaluation. The scarcity of essential supplies, such as masks and ventilators, made it difficult for surgeons to perform surgery and required provider improvisation. In addition, intensified workloads and persistent stress on surgical teams contributed to burnout and impaired quality of care [7].

Studies in this area showed that multiple perioperative recommendations were produced in a short period of time, and many of the proposals were conflicting and based on anecdotal evidence [4,8]. In particular, there are no generalized recommendations for surgical service delivery during pandemics. Surgical delivery must be incorporated into the WHO agenda for national health planning because of its transversal nature and synergistic effects on the health systems. As a consequence of the pandemic, patients are denied access to surgery, which can lead to irreversible functional decline and a poor prognosis. To continue providing surgical treatment during or after a pandemic, a backup plan for surgical services is necessary [9,10,11]. As we enter the post-pandemic period, ongoing monitoring and support for healthcare workers’ well-being remain critically important to address the residual effects of this prolonged public health emergency. The evaluation of strategies implemented during the pandemic to sustain surgical services should inform policies and protocols to strengthen the resilience of the healthcare system to potential future crises [3].

This scoping review, framed using the population, concepts, and context framework, aimed to evaluate the impact of COVID-19 on the utilization of elective, emergency, and essential surgeries. It examined the strategies implemented to optimize and sustain care delivery throughout the crisis. The purpose was to evaluate how COVID-19 affected surgical care, focusing on healthcare providers and patients within the surgical field (population). It explores the changes and adaptations in surgical practices and policies induced by the pandemic (concept), contextualized within the global COVID-19 health crisis, and its profound impact on surgical care delivery and management (context). The primary research question was: How has COVID-19 impacted surgical service utilization and what strategies were implemented to optimize care delivery throughout the crisis? Additional questions covered factors influencing changes across surgical areas, the effectiveness of hospital efforts to balance risks, resource and procedure prioritization, impacts of delays and cancellations, the evolution of perioperative guidelines, and lessons from providers for future preparedness. To answer these questions, this review synthesizes findings on changes in surgical services across specialties during the COVID-19 pandemic. The key factors and strategies are summarized. Dedicated sections examine the impact on general, cardiac, oncologic, orthopedic, head and neck, plastic, and pediatric surgeries.

## 2. Methods

**Figure 1:**
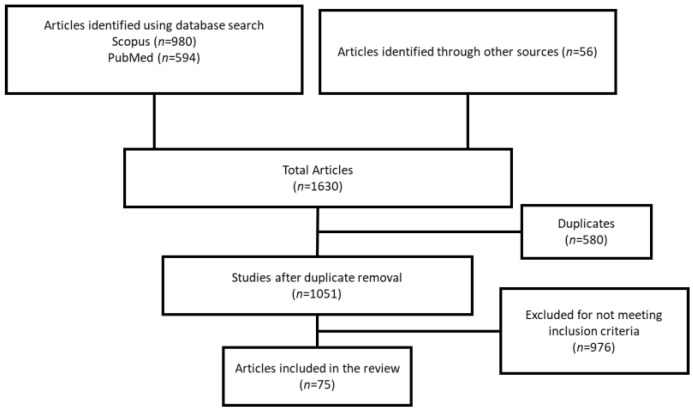
PRISMA flow diagram depicting systematic literature screening and selection.

A literature search was conducted using the preferred reporting items for systematic reviews and meta-analysis extension for scoping reviews (PRISMA-ScR) guidelines (Figure 1). A detailed search was conducted for articles and declarations that addressed surgical patient management during the pandemic. The PubMed and Scopus databases were searched on 29 January 2023. Searches were conducted for queries such as: (“surgical procedures, operative”[MeSH Terms] OR (“surgical”[All Fields] AND “procedures”[All Fields] AND “operative”[All Fields]) OR “operative surgical procedures”[All Fields] OR “surgical”[All Fields] OR “surgically”[All Fields] OR “surgicals”[All Fields]) AND (“facilities and services utilization”[MeSH Terms] OR (“facilities”[All Fields] AND “services”[All Fields] AND “utilization”[All Fields]) OR “facilities and services utilization”[All Fields] OR (“services”[All Fields] AND “utilization”[All Fields]) OR “services utilization”[All Fields]) AND (“efficiencies”[All Fields] OR “efficiency”[MeSH Terms] OR “efficiency”[All Fields] OR “efficiencies”[All Fields] OR “efficient”[All Fields] OR “efficiently”[All Fields] OR “efficient”[All Fields]) AND (“COVID 19”[All Fields] OR “COVID 19”[MeSH Terms] OR “COVID 19 vaccines”[All Fields] OR “COVID 19 vaccines”[MeSH Terms] OR “COVID 19 serotherapy”[All Fields] OR “COVID 19 nucleic acid testing”[All Fields] OR “COVID 19 nucleic acid testing”[MeSH Terms] OR “COVID 19 serological testing”[All Fields] OR “COVID 19 serological testing”[MeSH Terms] OR “COVID 19 testing”[All Fields] OR “COVID 19 testing”[MeSH Terms] OR “sars cov 2”[All Fields] OR “sars cov 2”[MeSH Terms] OR “severe acute respiratory syndrome coronavirus 2”[All Fields] OR “ncov”[All Fields] OR “2019 ncov”[All Fields] OR ((“coronavirus”[MeSH Terms] OR “coronavirus”[All Fields] OR “cov”[All Fields]))). Supplemental studies were identified by scanning the references of included studies.

The full text of the guidelines was obtained for potentially relevant records, and inclusion and exclusion criteria were applied. We only included articles and guidelines in English that provided (i) practical guidance on operative and perioperative management during the pandemic, (ii) utilization of surgical services during the pandemic, (iii) efficiency of the surgical system during the pandemic, and (iv) hospital policies and frameworks for the delivery of surgical care during the pandemic. Editorials, commentaries, and articles written in languages other than English were also excluded.

Two independent reviewers (NA and MH) screened the titles and abstracts of the retrieved records against the following eligibility criteria: (1) English language publications, (2) reporting on the delivery of surgical care during COVID-19, and (3) discussing practice changes, service volumes, efficiency impacts, policies, or surgeon perceptions. Relevant full-text articles were independently assessed for eligibility by both the reviewers. Disagreements were resolved by consensus. To ensure the uniqueness of the included studies and address potential duplications, we carefully screened titles and abstracts after removing duplicates. Relevant information from the included studies was extracted by two independent reviewers (NA and MH) according to the categories in a standardized form to enable evidence mapping, including (1) study characteristics, (2) reported changes in surgical services, (3) specialty-specific practice changes, (4) surgeon viewpoints, and (5) hospital policies. Extracted data were compared between reviewers to resolve discrepancies, and compiled into evidence tables mapping the current literature across these topics. Gaps and future research needs are highlighted based on a broad characterization of the existing literature.

## 3. Results and Discussion

A total of 1574 records were identified after the literature search, and an additional 56 articles were identified through other sources. After removing duplicates, 1050 studies were screened and 75 were eligible (Table 1 and Table S1). Among the included studies, 50 analyzed changes in healthcare use levels, 13 provided frameworks, four focused on factors influencing access and barriers, and eight studied multiple aspects. Table 1 summarizes the characteristics of key studies examining the impact of the COVID-19 pandemic on surgical services and care delivery, outlining study attributes such as author names, sample/data sources utilized, and highlighting notable findings.

These studies indicate that the COVID-19 pandemic caused major disruptions in surgical care and services globally. Numerous studies have documented sharp declines in surgical volumes across most specialties, resulting from postponed elective procedures and reduced efficiency [7,20]. Several studies provided guidelines and protocols aimed at facilitating the safe resumption of surgical services through protective measures and triage prioritization systems [12]. The rapid shift to telemedicine and virtual care was another notable trend, allowing remote pre- and postoperative management [6,35]. However, the impact on surgical training programs was profound, with significant reductions in hands-on education [31]. Furthermore, data on the effects on surgical outcomes are limited, although some studies reported worse results in certain cohorts of patients [33,34]. Overall, COVID-19 fundamentally altered surgical care delivery in settings worldwide, requiring new workflows, care models, and research on their lasting impact on patients and providers.

The results and discussion sections contain the following parts: guidelines and management approaches, impact on surgical service utilization with a general report subsection, followed by specialty-specific subsections on general, cardiac, thoracic, vascular, oncological, orthopedic, head and neck, plastic, and pediatric surgeries. This is followed by sections on the impact of COVID-19 on healthcare professionals, surgeons ‘ perspectives, and hospital policies.

### 3.1. Guidelines and Management Approach

Globally, health systems struggled to manage an increasing number of critically ill COVID-19 patients while maintaining essential surgical services and expanding their abilities to handle surgical cases delayed by the pandemic [39]. As the world progresses through successive pandemic steps and plans for future pandemics, it has become necessary to understand the impact of the pandemic on the utilization of surgical services and develop a decision-making platform that enables surgeons to remain adaptable to the needs of their patients while operating within a new healthcare framework. The following discussion outlines the guidelines and efforts made to develop a framework for managing surgical priorities during crises:

The European Association of Endoscopic Surgeons (EAES) and the Society of American Gastrointestinal and Endoscopic Surgeons (SAGES) published guidelines on surgical service delivery during the COVID-19 outbreak [12]. The EAES and SAGES guidelines emphasize surgical team safety, recommending minimal staff in operating rooms and advanced filtration for minimally invasive procedures to reduce the risk of virus spread. Universal personal protective equipment use was advised along with preoperative COVID-19 testing and designated operating rooms for infected patients. Whenever possible, it was recommended that all elective surgical and endoscopic procedures be postponed until the pandemic peaked. Patients should undergo surgery only if they have life-threatening problems or cancer with aggressive signs. This approach was believed to minimize the risk to the patient and healthcare team and the use of critical resources, such as beds, ventilators, and personal protective equipment. Additionally, it was recommended that all nonessential personnel be able to work from home and that only a small number of people be involved in face-to-face decision-making. All non-emergency clinics and patient appointments were rescheduled and conducted remotely via telephone or video conferencing. All meetings involving multiple disciplines should be conducted digitally and be restricted to team members. The intraclass correlation coefficients of the included institutions confirmed “acceptable” inter-institutional reliability. Given their experience, the authors believe that MeNTS is superior because it uses quantitative rather than subjective decision making [28]. Reflecting the positive impact of the MeNTS format, an Ethiopian tertiary care center established a plan-do-study-act model that reduced preoperative waiting time, lowered cancelation rates, and increased monthly inpatient bed utilization during COVID-19 [40]. Although the MeNTS framework offers valuable guidance in prioritizing medically necessary and time-sensitive procedures, its application is not without challenges. Critics have pointed out that the subjective interpretation of these criteria can vary among surgeons. Additionally, the rigidity of the framework sometimes struggles to adapt to the rapidly changing landscape of the pandemic, posing challenges for real-time surgical decision making. Acknowledging these limitations is crucial for a balanced understanding, and highlights the necessity for the ongoing refinement of such frameworks.

**Figure 2:**
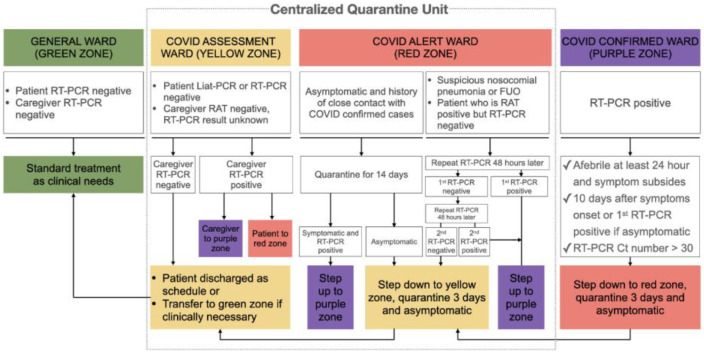
Admission flowchart for screening in centralized quarantine units [17]. Licensed under CC BY 4.0.

Wee et al. proposed a risk stratification method to allow rapid detection and exclusion of possible COVID-19 infections in surgical patients, thus safeguarding surgical service consistency during the outbreak. If emergency procedures were required before segregation, patients were treated in the same way as the suspected cases of COVID-19, with suitable protection. Risk-based screening, diagnosis, and isolation of surgical patients with COVID-19 ensures the continuity of surgical services [15]. In Taiwan, patients who required surgery were assigned to the appropriate wards using a four-level classification system (Figure 2). This was applied to patients undergoing elective surgery who visited the outpatient department and emergency surgery patients who arrived at the emergency department [16]. After isolation, patients with suspected or confirmed infections may be transferred to less-stringently monitored wards. During this procedure, the number of surgical patients in the emergency department (ED) on a particular day was positively correlated with the occupancy ratio of the central quarantine unit. Acute care surgical services and quarantine capabilities were maintained throughout the COVID-19 pandemic owing to the admission strategy [17].

Enhanced recovery protocols (ERPs) are the best-practice evidence-based recommendations used throughout the perioperative continuum to reduce postoperative anxiety, prevent problems, and accelerate healing. However, the standardization and optimization of ERP during this high-risk period were necessary to reduce the likelihood of unfavorable outcomes due to weaknesses in the provider, patient, and system. Additional provisions were recommended for patient instruction, infection testing, rehabilitation, intraoperative infection, and risk reduction for thromboembolism in light of the COVID-19 pandemic [41]. Clinical care regimens, such as ERP, are highly dependent on the monitoring of patient results and treatment changes [41]. Enhanced recovery after surgery (ERAS) is an international program that permeates the perioperative care sector and aims to improve the surgical outcomes. Surgery and anesthesia have reached new heights, and the outcomes of patients and health systems have improved because of this global crisis [42]. An innovative pilot weekend surgical quality improvement initiative, operating room ramp-up after COVID-19 lockdown ends–extra lists (ORRACLE-Xtra), was designed and implemented to improve patient access to surgery, operating room efficiency, and parental and staff satisfaction. It was implemented by an academic pediatric tertiary care facility using the define, measure, analyze, improve, and control framework. COVID-19 hospitals saw a 5% surgical backlog reduction using ORRACLE-Xtra [43].

### 3.2. Impact on Surgical Service Utilization

#### General Report

**Figure 3:**
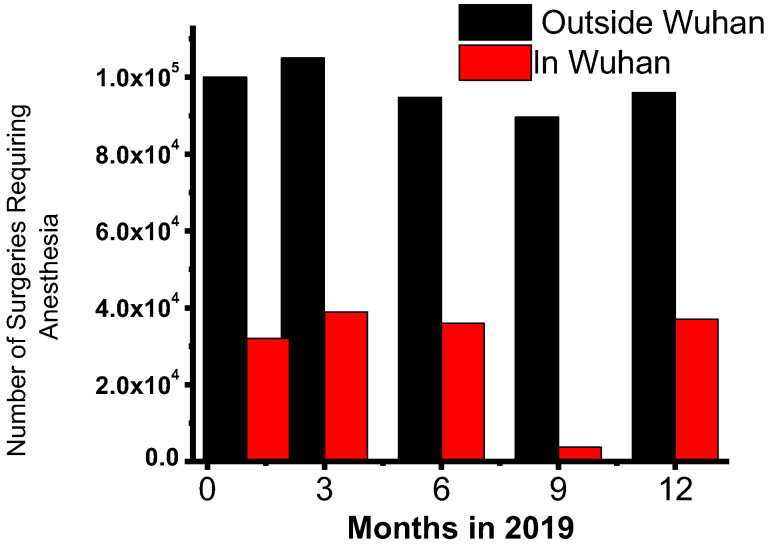
Monthly surgical cases requiring anesthesia from January 2019 to December 2019 in Hubei Province, China. Data were collected from 29 hospitals in Wuhan (which implemented a lockdown from January 23 to April 8, 2020) and 197 other hospitals in Hubei. This shows dramatic declines in surgical volumes during the first months of the COVID-19 outbreak, especially in Wuhan, highlighting the impact of public health emergencies on the delivery of surgical services. Adapted from Wu et al. 2021 [5].

In February 2020, the number of surgical cases in Wuhan, the epicenter of COVID-19, decreased by more than 90% compared with the previous year (Figure 3). The city lockdown aggravated this loss, leading to a sharp decrease in processes and severely delayed recovery [5]. In the United States, COVID-19-related reasons for suspending elective surgery are expected to cost approximately USD 20 billion in lost income [44]. The total number of surgical operations performed in England and Wales in 2020 indicated an almost 30% decrease in national surgical activity. Emergency surgical operations were reduced by 13.4%, while elective surgical procedures were reduced by 38.6%, leading to more than 1.5 million canceled operations [7]. A Canadian study used time-series analysis to estimate the impact of COVID-19 on surgical services using networked health administrative datasets. In particular, 255,501 surgical procedures were performed prior to the implementation of the COVID-19 protocols in 2020. Only 30,033 surgical procedures were performed during the COVID-19 pandemic. Compared to 2019, the weekly rate of surgical operations did not drop considerably after week one but was reduced by 78% by week 2 and 83% by week 3. As expected, ambulatory procedures experienced the most significant decline compared with 2019. The provincial order did not mention urgent procedures, yet they were reduced by 36% and 49% in weeks two and three, respectively [20].

Preoperative involvement changed from 89.8 percent in persons before shelter-in-place restrictions to 70.2 percent telemedicine between 1 January 2019, and 13 June 2020, according to a study focusing on referrals to surgical services, concluding that telemedicine allows surgeons to provide preoperative and postoperative treatment in a time-efficient and cost-effective manner [35]. During the pandemic, the New York City Hospital developed an auxiliary central catheter emergency support service to facilitate the movement of surgical personnel, provide learning opportunities for trainees, and improve the efficiency of critical care teams. This service contributed to the implantation of more than 100 invasive catheters with a low risk of complications, saving each patient for at least 30 min during surgery [45].

In a retrospective observational analysis using the RedCap ACS COVID-19 registry, it was reported that more than 50% of patients who did not undergo surgery could return home compared to less than 40% of those who received surgery. In general, surgery significantly affects the discharge destination of patients with COVID-19 [46]. Retrospective claims data from a national healthcare technology clearance house were used in a cohort study to describe the growth or decline in the number of surgical procedures performed in the United States. The main categories of procedures, subcategories, and exemplar operations were used to examine surgical procedures throughout the continuum from elective to emergency. Studies assessing surgical volumes during COVID-19 shutdowns and re-openings have revealed significant impacts as well as the adaptability of healthcare systems. An analysis of a state-wide medical system found that compared to prepandemic baselines, procedures decreased dramatically during the initial lockdown period, with otolaryngology and ophthalmologic surgeries exhibiting the largest proportional declines. By quantifying the overall reduction during the shutdown alongside specialty-specific variances, these data highlight the sudden constrained capacity for all elective operations, reflecting systems overwhelmed by the acute pandemic response. However, despite overlapping with the highest number of regional hospitalizations for COVID-19, surgical volumes recovered to baseline levels after reopening. The ability to restore pre-outbreak surgical capacity, even amid ongoing pandemic strains, demonstrates the resilience of providers and administrators to adapt policies and protocols to balance competing demands. However, deferrals also accumulate into substantial backlogs, underscoring the need for continued flexibility in efforts to resume routine surgical services [47].

Multiple studies have quantified the decline in operating room utilization during the initial lockdowns. For example, Low et al. reported that OR usage rates dropped from 66% before the pandemic to 52% after implementing preparation policies, along with sharp reductions in elective surgeries and outpatient work [48]. Consequently, the wait times for consultations and operations have drastically increased. These impacts spanned emergency and elective surgeries across specialties. Another study revealed a decrease of 38% in trauma and 57% in emergency general surgeries during COVID-19 surges [49]. However, another study found that patients hospitalized during the peak months had higher mortality, suggesting care limitations [50]. Analyses have also demonstrated changes in case mix and patient profiles [51]. In Ontario, patients with prior COVID-19 infection had a lower risk of multiple common surgeries [52]. An assessment of 10 hospitals in Spain revealed decreased laparoscopic approaches and longer stays, indicating that the standards had suffered [53]. Studies have also projected increased costs due to surgical delays [29]. A significant reduction in ED admissions was reported in Portugal. During the COVID-19 pandemic, approximately 30% of patients underwent urgent/emergency surgery. Waiting time for surgery was not significantly different between the groups. However, patients who underwent surgery during the 2020 pandemic had higher mortality rates than those who underwent surgery in 2019. A reduction in surgical volume is correlated with an increasing number of infected cases [54]. Another study examined the impact of surgical services at a major hospital in South Africa during the first few months of the COVID-19 pandemic. The results demonstrated that operating room caseloads decreased substantially, with an overall 30% reduction in surgical volume compared with the pre-pandemic levels. However, intensive care unit admissions remained constant despite the drop in surgeries performed. Furthermore, the number of cancelations decreased proportionally with a decrease in the total number of cases. Researchers estimated that performing four additional procedures per day for 315 days would be required to remove the backlog generated during the initial 4 months of disruption. This study quantified the sudden constraint in surgical capacity at a single center during the onset of a public health crisis. It also highlighted the increasing demand for delayed essential procedures, underscoring the challenges hospitals face in managing new obstacles to care delivery posed by the pandemic [55]. The COVID-19 pandemic required rapid adoption of telemedicine to maintain continuity of care when in-person visits were restricted. The SmartDoc project, an initiative in Italy, was designed to enable the ongoing treatment of lung cancer patients through virtual consultations. This national program worked within the existing regulatory system to authorize and reimburse video visits. An analysis of SmartDoc found that 70% of the patients who used telemedicine services reported being highly satisfied. Most participants preferred the video format and rated it as comparable to or better than the traditional in-clinic consultations. This rapid transition to remote care delivery highlights how regulatory flexibility and technology implementation can increase access to health care during public health crises. This also demonstrates the potential of telemedicine to facilitate safe and effective virtual treatment when the traditional options are limited [6]. Table 2 summarizes the significant research findings demonstrating changes in surgical services during the COVID-19 pandemic across specialties and care settings.

### 3.3. General Surgery

In the Western Cape Province, SA, a comparison of the volumes and proportions of general surgery operations was conducted in 2019 and 2020 in the six districts and at the regional level. In general, the number of surgical procedures decreased by 44% between 2019 and 2020, with a 46% decrease in elective procedures, 22% decrease in emergency procedures, and 42% decrease in trauma procedures. However, the numbers of emergency appendicectomies and cancer surgeries did not decrease. The authors stated that if every hospital could perform one more surgery per day, the surgical backlog of elective procedures 4 months later would be cleared within 4–14 months [50]. The number of elective laparoscopic cholecystectomies performed in the United Kingdom decreased during the COVID-19 outbreak. Isolated day-case units were suggested as COVID-19-cold operating locations, allowing surgical operations to restart [56]. In Brazil, the number of kidney transplants performed per million individuals decreased by 23.9% during the pandemic [57]. The numbers of people on the pandemic waiting list and those who died while waiting were 3.6% and 36.8%, respectively.

Acute appendicitis is a common surgical emergency affecting people worldwide. In Thailand, the overall number of acute appendicitis cases was reduced by 13.4% [58]. This decline was primarily driven by a reduction in the incidence of simple acute appendicitis. However, the pandemic had only a slight influence on the frequency at which generalized peritonitis was diagnosed. Since the frequency of generalized peritonitis and its sequelae remained stable despite fewer admissions during the COVID-19 lockdown, Sukmanee et al. recommended that the prevalence of acute appendicitis in Thailand be overstated [58].

A worldwide survey of urologists examined the impact of COVID-19 on urological care delivery. The results revealed strained resources, with 40% of respondents reporting positive staff cases, and more than a quarter noting personnel shortages or redeployment to pandemic duties. Only one-third felt that they had adequate protective equipment, causing many people to feel unsafe at work. The pandemic reduced the demand for urological services such as clinic visits, exams, and surgeries worldwide. The decline worsened with higher regional severity of COVID-19. Non-cancer services experienced greater disruption than oncology care. These data highlight the workforce and logistics challenges constraining the urological capacity during the pandemic. They also underscore key differences in the impact of pandemics depending on the urgency of the service [59]. Another study analyzed the changes in urological department activities during a 21-day pandemic response period. The total number of admissions decreased by more than a quarter, driven by a 32% reduction in elective surgeries compared with a 30% increase in acute procedures. Despite a 32% decrease in total outpatient consultations, virtual phone consultations sharply increased by 274%, highlighting the rapid expansion of telehealth services. However, the procedural clinics saw an 85% drop, reflecting deferred care for nonurgent needs. They estimated substantial savings in patient travel due to the use of telemedicine. These data reveal shifting priorities and capacities within urological care during the COVID-19 peak, including the growing demand for telehealth that allows access despite limitations [60].

### 3.4. Cardiac, Thoracic, and Vascular Surgery

An analysis of all cardiac surgery patients before and after the lockdown revealed that more than 60% of the surgeries were urgent/emergency procedures, and up to 39% before the lockdown. Sternal wound infections can be avoided by strict adherence to recommendations throughout the perioperative phase [32]. A UK study of aortic valve surgeries in a two-site center revealed a significant initial impact of COVID-19. The total operations decreased by 38%, driven by a 70% drop in electives, compared with a 159% increase in urgent and emergency cases. The pandemic forced the deferring of lower-risk procedures, while urgently managing more complex cases. Consequently, the analysis found a significantly higher attendant surgical risk in 2020 than that in 2019. These data quantify the dramatic changes in cardiac surgery priorities and patient acuity resulting from COVID-19 care interruption. It highlights how managing only the most critical cases risks heightened complications, underscoring the need to maintain greater capacity [61]. Patients who underwent open or endovascular thoracic aortic procedures in one of three tertiary cardiology centers were included in a prospective study that collected data over two time periods. Compared with patients who underwent surgery in 2019, those who underwent surgery in 2020 had a significantly higher median EuroSCORE II [34].

Several studies have examined the effects of COVID-19 on vascular surgeries. A review of a quality improvement platform revealed a decreased volume of institutional cases during the pandemic [62]. Another analysis found that in 2020, 61% of patient encounters occurred via teleconsultation, while outpatient clinic procedures decreased by 46% annually [36]. The authors noted that telehealth could maximize the efficiency of care for vascular surgery patients, both during and after the pandemic. Furthermore, studies focusing on patients with peripheral arterial disease showed more severe limb ischemia presentations, requiring urgent inpatient treatment during the COVID-19 peak [33,37,63]. The analyses also found that telemedicine kept vascular patients safe while reducing travel burden during care interruptions. Overall, this evidence demonstrates the effects of COVID-19 on redirecting vascular surgery priorities to more acute cases while highlighting the potential of telehealth to safely expand access and efficiency if integrated thoughtfully.

### 3.5. Oncological Surgery

Sutjiadi et al. examined referrals to specialized clinics for oncologic surgery at an academic tertiary care facility after California implemented stay-at-home orders within the same timeframe the previous year. The severity of the diagnosis, insurance coverage, time from referral to the first appointment (TRFA), and the total number of surgical consultations were evaluated. A 20% decrease in the number of patients was observed between the two periods. Significant variations were observed in the percentage of patients who underwent surgical and thoracic oncology visits, Medicaid insurance, and suspicious and malignant diagnoses during the COVID-19 pandemic. The TRFA was often lower during the COVID-19 period. When stay-at-home orders were introduced, patients with greater acuity and vulnerability were observed in oncological surgical specialty clinics [64]. At Massachusetts General Hospital, a same-day strategy for mastectomy and rapid breast reconstruction proved successful, with no patients requiring readmission, emergency department (ED) visits, or having postoperative complications [65].

### 3.6. Orthopedic

Researchers from South Africa analyzed data from the prior periods before COVID-19 and COVID-19 in 2020 on the number of orthopedic surgery cases, wait times for emergency room patients, outpatient clinic visits, admissions to the ward, bed occupancy and total days of hospitalization. The hardest imposed month of lockdown, April 2020, was found to be the most affected, according to the authors, even if the number of surgical cases had steadily decreased over the three months before. The overall number of hospital admissions, outpatient visits, and operations decreased by more than half in April 2020 compared with April 2019 [25]. In a study of surgical trends in the United States, Khan et al. found a general 14% decrease in the number of shoulder arthroplasty procedures performed per 1000 Medicare members. The transition to an outpatient surgical paradigm could be carried out safely and effectively, and could be potentially beneficial because of the reported reduction in length of stay (LOS) and the increase in discharged home rates, with no discernible change in readmission at 30 days of hospitalization [26].

Using prospectively gathered data on theater timings and procedures, Sharkey et al. investigated theater efficiency in the UK and found a considerable decrease in the number of points in each session. They also observed that owing to COVID-19, the efficiency of the theater was poor, and the conformity of preoperative segregation was low, indicating the need for more techniques to improve elective orthopedic care [21]. Another study examining the effect of COVID-19 on theater utilization in the UK reported that although the number of patients in the first COVID-19 wave remained relatively stable between 2019 and 2020, the transfer time increased dramatically, while the median surgical preparation time and operating time were not statistically significant [22]. Another study in the UK found that the number of new admissions in 2020 was approximately 20% lower than that in 2019. Phase 1 of COVID-19 also showed a decrease in all trauma subspecialties, but hip fractures accounted for more than half of all trauma cases, with a shorter mean stay and longer time in the theater [66]. The effect of SARS-CoV-2 infection on operating room efficiency in London’s four busiest trauma centers was studied by Jeyaseelan et al. They found that although the frequency of open reduction and internal fixation surgeries decreased in 2020, the number of orthopedic patients increased. The median time between sending a patient and their arrival in the anesthetic chamber increased significantly [67].

A multinational survey by Hall et al. revealed a drop in the quality of orthopedic services in different centers in 11 countries [68]. Involuntary staff turnover, transfer of inpatient sections, and reduced availability of operating rooms were the main causes of this drop. The numbers of orthopedic surgeons, physical therapists, and orthopedic trained nurses were reduced by half, one-third, and two-thirds, respectively. With the relocation of inpatient facilities, the time spent moving patients and personnel between different therapeutic settings increased. More than half of the centers reported a reduction of more than 50% in operating room access; 80% of the centers reported a loss in theater efficiency due to delays in preoperative COVID-19 testing and PPE usage, personnel and resource reallocation, and prolonged anesthesia and transfer times [68]. Montanari et al. examined the HUB facility for hand surgery and microsurgery in Emilia-Romagna [23]. They found that the overall surgical activity was reduced by approximately 70%, with a notable loss of approximately 40% in elective surgery and a slightly smaller decline of >35% in urgent surgery. Additionally, concerns about risk management and legal accountability significantly limited the use of telemedicine [23].

### 3.7. Head and Neck

Healthcare workers are more susceptible to COVID-19 transmission during head and neck surgical procedures because of aerosolization of virus particles from the nasal mucosa and mouth cavity [18]. Therefore, patients with facial fractures have distinct difficulties owing to the wide range of injuries and the need to take precautions against infection. Most patients who undergo facial fracture surgery are at a higher risk of contracting COVID-19 because of the proximity of the surgical site to the oral cavity and naso-oropharyngeal mucosa as well as the potential for viral particle-containing secretions to aerosolize. Hsieh et al. used an algorithm to determine a balance between sensible healthcare use, patient protection, and patient and medical personnel safety. Depending on the latest best practices, viral transmission statistics, and an acceptable amount of PPE, facial trauma surgeries were prioritized based on their urgency [19].

According to another study, reducing the amount of time that patients are exposed to aerosolized secretions during surgery is the most important step in reducing the risk of contracting COVID-19. Based on the surgeon’s experience, this was achieved with an average incision-to-cuff inflation time of less than five minutes. Fogging is the biggest obstacle to PPE tracheostomy as it can make the surgeon uncomfortable and make the process more difficult. Antifogging medications could help, but the experience of surgeons is crucial because they utilize two additional senses: intellect to detect any decrease in oxygenation and kinesthetic awareness to palpate the trachea at every step [69]. In the context of COVID-19, Krishnamoorthy et al. reported concerns regarding bedside tracheostomy by finding that it does not cause further damage to patients if performed two weeks after intubation. Importantly, the findings indicate that bedside tracheostomy is safe for proceduralists to perform within this period, and that the tracheostomy method does not affect patient outcomes [70].

To determine the efficacy and safety of drain removal at home, Sethia et al. conducted a survey of patients who underwent head and neck surgery. Almost 90% of drains that were successfully removed without major problems were located in the neck region. During the COVID-19 pandemic, it was discovered that the removal of home drains not only helped save money but also prevented potentially hazardous in-person encounters between patients and healthcare providers [71]. The feasibility and applicability of telemedicine in rhinology were investigated in a study conducted in Saudi Arabia. The overwhelming majority of participants said that they were satisfied with the services they received. The findings demonstrated that telemedicine could effectively manage and screen rhinology cases during public health emergencies while maintaining a high level of patient and practitioner safety [38].

### 3.8. Plastic Surgery

A recent cross-sectional analysis examined the impact of COVID-19 on plastic surgery in the United Kingdom (UK). Operating and outpatient capacities were significantly reduced. Anesthesia practices were adapted with the increased use of local anesthetics for hand procedures. Surgeries were shorter, with fewer microsurgeries, whereas breast reconstruction was nearly stopped. Changes in practices varied regionally. Widespread redeployment occurred, although telemedicine allowed continued patient management and training despite its limitations. In summary, this study highlights the multifaceted effects of the pandemic on plastic surgery delivery. This reveals how practices judiciously adapt standards of care to constraints, while leveraging virtual platforms to maintain training and access. The flexibility and innovation of plastic surgery teams during COVID-19 could inform approaches to surmounting care disruptions [24].

### 3.9. Pediatric Surgery

Studies have revealed varying effects of COVID-19 on pediatric surgery. A tertiary hospital analysis found a 55% overall decline in procedures requiring anesthesia, with disproportionate reductions between inpatient and outpatient surgeries [61]. Another study reported a 40% decrease in pediatric fractures, although with increased time to subspecialty follow-up [72]. Researchers have also observed changes in ED care models during the pandemic, including more procedures for injuries, shorter stays, and increased appendicitis severity [30].

Beyond direct care, a survey of more than 300 pediatric urologists worldwide showed that COVID-19 drove a 75% increase in the use of telemedicine [73]. However, analyses have found that minorities have lower utilization of telehealth, which requires investigation to ensure equitable access [74]. Additionally, the incorporation of telehealth was shown to reduce emissions, highlighting environmental benefits during care disruptions. Table 3 summarizes notable research evidence regarding the changes in surgical care during the COVID-19 pandemic across various specialties.

### 3.10. Impact of COVID-19 on Healthcare Professionals and Hospital Policy

#### 3.10.1. Health Care Professionals and Surgeons’ Perspectives

The COVID-19 pandemic has profoundly disrupted the delivery of surgical services worldwide, necessitating major adaptations with multifaceted effects on the healthcare workforce. The analyses estimated that most surgeon absences were unlikely to significantly constrain elective surgery capacity alone during outbreaks [75,76]. However, extensive surveys in hospitals revealed severe reductions in access to elective, cancer, and even emergency operations. One study surveyed 133 surgeons across 85 hospitals in South Africa to assess the impact of the COVID-19 pandemic on their surgical services [77]. The results demonstrated extensive disruptions, with the vast majority of hospitals cancelling elective procedures (99%) and over half curtailing emergency operations (54%). Additionally, one in three hospitals converted operating rooms to critical care units to accommodate COVID-19 patients. Outpatient visits saw major reductions, and surgical beds and staff were reallocated to pandemic response efforts. The intended goal was de-escalation to conserve resources; however, the long-term effects may include increased backlogs and negative health outcomes in patients undergoing delayed surgery. Overall, the survey highlighted how surgical capacity was diminished to focus on the COVID-19 crisis in South Africa, compromising access to essential care [77].

Frontline surgeons also reported concerns about perceived suboptimal institutional strategies to protect staff from infection risks [78]. Conflicting guidelines on approaches, such as laparoscopic surgery in patients with COVID-19, further complicated delivery [56]. However, most surgeons worldwide continued to perform urgent operations on infected patients despite the overall decrease in surgical volume. Enhanced communication, leadership, workforce planning, training, and regulatory compliance have been identified as critical needs for improving pandemic management [79].

The rapid expansion of telehealth has enabled continuity of care despite in-person restrictions [3,80]. More than 85% of surgeons surveyed across Australia participated in virtual consultations during the pandemic, most wishing to continue access after COVID-19. Although a majority of them found telehealth satisfactory for at least half of their consultations, only 38% viewed it as equivalent to in-person visits [80]. The inability to conduct physical examinations and limited capacity to convey serious news or address conflicts were common concerns [80]. Overcoming medical, technical, and financial obstacles is imperative to fully realize the benefits of telehealth while balancing its limitations in surgical care [80]. Assessing the experience of frontline surgeons provides an invaluable perspective to guide policy responses. Ensuring adequate workforce protection, updating practice guidelines, improving leadership coordination, and thoughtfully integrating virtual care solutions are the key priorities emerging from this global disruption to surgical services. Table 4 summarizes the research findings on changes in surgical practices during COVID-19 as perceived by frontline surgeons across domains, such as surgical volumes, workforce deployment, use of telehealth, and pandemic management needs.

#### 3.10.2. Hospital Policy

**Figure 4:**
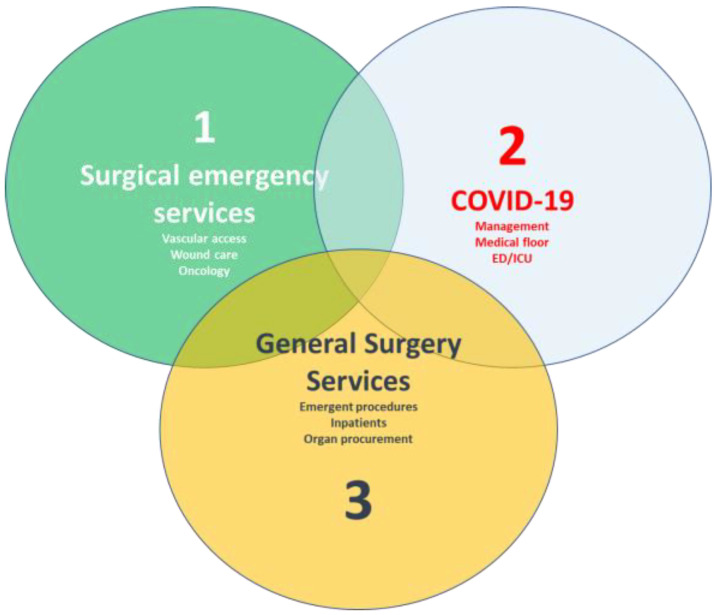
Core components of surgical service utilization.

Hospital policies have played a critical yet complex role in balancing infection risks while sustaining care delivery during the pandemic. In Singapore, hospitals mobilized resources and enacted measures even before the first case emerged, including postponing non-urgent procedures, establishing specialized COVID-19 wards, implementing geographic segregation within clean wards, and alternating teams to allow adequate rest [48]. These proactive steps were intended to maintain essential services, while preventing intra-hospital spread. In the US, stopping nonemergent elective surgeries rapidly became widespread based on guidelines from the American College of Surgeons (Figure 4) [31]. However, surgical residents were often redeployed to assist overloaded services, which was made possible by shifting graduate medical education policies to pandemic emergency status [31]. This allowed flexibility to meet the first-line needs while maintaining duty-hour regulations.

In Saudi Arabia, a study revealed that policymakers promoted digital health solutions to improve access to care despite disruptions [81]. However, citizens reported problems with booking medications or equipment online and a lack of personalized virtual care, highlighting the disparities in utilization between the groups that policies must address. In Australia, more than 85% of the surgeons surveyed engaged in new virtual consultations during the pandemic, although many desired continued access post-COVID-19 [80]. Overcoming medicolegal, technical, and financial barriers will be the key to supporting long-term integration.

Reviews of hospital pandemic plans identify common policy domains, such as staff training on updated safety protocols, reducing non-urgent visits and procedures, establishing team-based emergency administration, and recognizing and managing COVID-19 infections in patients. However, they emphasize that policies require context-specific tailoring and continuous updates as evidence rapidly emerges [82]. For example, better protection is required for endoscopic procedures that generate respiratory droplets than for low-risk surgeries. Multidisciplinary involvement, structured surgical risk assessment, and clear communication are vital for successful, tiered case reduction [9]. Post-pandemic surgical backlogs appear unavoidable and require effective policies to minimize the adverse outcomes of delayed essential care.

All of these studies underscore the critical but complex role of hospital policies during public health crises. Although many policies, such as stopping elective surgery, were widely adopted, one-size-fits-all approaches often proved insufficient. Innovations such as telehealth integration may persist after the pandemic but require context-specific planning to address disparities. Ultimately, through thoughtful guidelines developed through stakeholder involvement, hospitals can balance their constraints with safe and sustained delivery. Table 5 provides a summary of research evidences on hospital policy responses across key domains during the COVID-19 pandemic, including changes to elective surgeries, workforce protocols, infection control, digital health expansion, telehealth integration, guideline updates, risk assessment incorporation, and backlog management.

## 4. Variability in Existing Evidence

The studies included in this broad scoping review encompassed a heterogeneous set of articles that varied substantially in context, study design, patient population, data sources, and methodological rigor. While expected, given the exponential growth of pandemic-related literature, such variability presents barriers to clean evidence mapping and signals the need for more consistent and focused systematic reviews. Apparent conflicts emerged across the study findings even within similar specialties. For instance, while several studies reported sharp declines in cancer surgeries during the early months of the pandemic, some articles noted stable or even increased volumes attributed to concerted efforts by institutions to continue cancer care. Determining the reasons for these differences warrants further investigation. Furthermore, beyond volume changes, the assessments of efficiency metrics, practice modifications, policies, and surgeon experience diverged significantly between the articles. This scope and discordance in the existing literature revealed gaps for further research into why such variances manifest themselves. Therefore, a context-specific analysis is imperative. By highlighting these evident variabilities and conflict issues in the current body of evidence, we emphasize the need for rigorous systematic reviews that concentrate on precise questions within defined settings and specialties. This will enable a clearer determination of the reasons for the divergent findings and biases versus true setting-specific differences.

## 5. Limitations

This scoping review has several limitations. This review included only studies published in English, excluding potentially relevant findings in other languages. It was restricted to peer-reviewed articles, omitting grey literature, preprints, conference proceedings, and other non-peer-reviewed sources with additional potential insights. Most of the included studies used retrospective data and methodologies, limiting the ability to infer causal relationships between the pandemic and outcomes. Most studies have focused on early pandemic timeframes with less data on the long-term impacts that are still evolving. The generalizability of the findings across regions may be limited, as most studies focused on high-income countries with advanced healthcare. The variability in how studies define and report utilization metrics inhibits cross-study comparisons and synthesis. Publication bias may have favored the reporting of significant and non-significant results. Rapid vaccine development and rollout were not accounted for in most studies, which may have influenced the volume and practice. As a scoping review, quality appraisal of the included studies was not undertaken, which should be considered when interpreting the evidence base. Further high-quality research addressing these limitations would be valuable for better understanding the global impact on surgical care. Researchers should focus on longitudinal analyses, standardized metrics, underrepresented regions, and emerging practices as the pandemic continues to evolve.

## 6. Recommendations

Based on the analysis in this review, several recommendations can be made to optimize surgical care delivery during future public health crises. Hospitals should develop context-specific pandemic plans to maintain essential surgeries through coordinated resource allocation and evidence-based protection of infection controls. Structured risk stratification systems should be implemented to guide the judicious triage of time-sensitive procedures by weighing factors such as potential postponement harm and disease transmission risks. Expanded training is needed for surgical teams on updated safety protocols, crisis care principles, and effective use of telehealth platforms to diversify care options in the face of restrictions. Monitoring and addressing care disparities exacerbated by delivery disruptions should be prioritized through centralized tracking and quality improvement initiatives. Research quantifying the long-term patient impact of pandemic-related surgical delays is essential to guide the national healthcare policy. These recommendations can help to strengthen the resilience of essential surgeries through inevitable future crises.

## 7. Conclusions

This systematic review aimed to elucidate strategies for optimizing and sustaining surgical care during public health crises such as the COVID-19 pandemic. An analysis of the literature reveals significant yet heterogeneous impacts on delivery across specialties and settings. The decline in elective surgeries frequently exceeded that in emergency and urgent procedures. However, new obstacles related to personnel, resources, and patient risks have emerged when caring for these complex cases. Thoughtfully designed hospital policies and procedural prioritization frameworks have proved vital for balancing infection control with equitable access. Support for frontline healthcare professionals through rapid practice changes and mental health impacts is also imperative.

## Figures and Tables

**Table 1:** Summary of study characteristics.

Category	Authors	Sample/Data Source	Key Findings
Guidelines for safe surgery during COVID-19	Francis et al. (2020) [12], Frakes et al. (2020) [13], Ksenak et al. (2020) [14], Wee et al. (2020) [15], Wake et al. (2021) [16], Hsu et al. (2021) [17], Chan et al. (2020) [18], Hsieh et al. (2020) [19],	Literature reviews, recommendations	Provided protocols and protective measures for safe surgical practices during the pandemic
Impact on surgical volumes and efficiency	Dobbs et al. (2021) [7], Gomez et al. (2021) [20], Sharkey et al. (2020) [21], Karanjia et al. (2021) [22], Leti Acciaro et al. (2020) [23], Johal et al. (2020) [24], Waters et al. (2020) [25], Avant-Garde et al. (2021) [26]	Surgical data from hospitals	Documented declines in surgical volumes and OR efficiency across most specialties
Surgical triage and prioritization	Prachand et al. (2020) [27], Saleeby et al. (2021) [28], Rovers et al. (2020) [29], Sullivan et al. (2020) [30]	Literature reviews, protocol development	Proposed frameworks for surgical triage and priority scoring systems
Adapting surgical training	Edwards et al. (2021) [31]	Surgical training programs	Disruptions to surgical education and hands-on training
Impact on surgical outcomes	Hussain et al. (2021) [32], Veraldi et al. (2022) [33], McPherson et al. (2021) [34]	Patient cohorts and outcomes	Limited data available, but some worsened outcomes observed
Telemedicine and virtual care	Kuehner et al. (2022) [35], Pardolesi et al. (2022) [6], Al-Thani et al. (2021) [36], Chen et al. (2022) [37], Alshareef et al. (2021) [38]	Telemedicine utilization data, patient surveys	Increased adoption of telehealth for pre- and postoperative care

**Table 2:** Highlights of evidence of changes to surgical services during the COVID-19 pandemic.

Study	Country	Year	Key Findings
Low et al. [48]	Singapore	2020	OR utilization down 14% during pandemic preparation policies; sharp drops in elective surgery and outpatient work.
Bugaev et al. [49]	United States	2020	38% decrease in trauma surgeries, 57% decrease in emergency general surgery during COVID-19 surges.
Chu et al. [50]	South Africa	2021	30% drop in surgical volume; proportional drop in cancellations.
McLean et al. [51]	United Kingdom	2020	Post-lockdown surgery patients were older, frailer, higher risk of cancer/obstruction.
Welk & Richard [52,53]	Canada	2021	Lower risk of common surgeries in COVID-19+ patients; decrease in kidney stone and gallbladder surgeries.
Guadalajara et al.	Spain	2021	Decreased laparoscopic approaches, longer hospital stays during pandemic.
Rovers et al. [29]	Netherlands	2022	Total hip replacement had greatest quality of life impact due to pandemic surgical delays.
Sá et al. [54]	Portugal	2021	30% drop in surgical volume but no change in waiting times; higher mortality during pandemic surgeries.
Laäs et al. [55]	South Africa	2020	30% drop in surgical volume; proportional drop in cancellations.
Pardolesi et al. [6]	Italy	2022	70% patient satisfaction with video consultations for lung cancer care during the pandemic.

**Table 3:** Evidence summary of changes in surgical care during the COVID-19 pandemic by specialty.

**Surgery Type**	**Key Findings**	**References**
General	Surgical volumes decreased substantially across districts and procedures, with electives and trauma down by around half but emergency appendicectomies and cancer surgeries maintained. Backlogs estimated at months with projections that increasing capacity by one additional case daily could clear in 4–14 months.	[50,56,57,58,59,60]
Cardiac	Urgent cardiac cases rose over 150% while electives dropped 70%, with attendant surgical risks increasing significantly.	[32,61]
Thoracic	Thoracic aortic surgery patients had higher severity levels.	[34]
Vascular	Vascular teleconsultations increased 61% though procedures decreased 46%, with more acute limb ischemia but telemedicine showing potential to mitigate burdens.	[36,37,63]
Oncologic	Oncological consultations decreased 20% with greater acuity and vulnerability during stay-at-home orders, but same-day mastectomy strategy proved successful, with no readmissions, ED visits or complications.	[64,65]
Orthopedic	Orthopedic surgeries and admissions dropped over 50% during peak lockdowns with 14% fewer shoulder replacements, longer theatre times but increased discharge rates. Multinational survey revealed halved orthopedic staffing and over 50% operating room reductions.	[21,22,23,25,26,66,67,68]
Head and Neck	Facial trauma surgeries prioritized by urgency balancing transmission risks with time minimization key - under 5 minutes from incision to cuff inflation. Home drain removal successfully implemented for most neck surgeries, preventing hazardous in-person encounters while telemedicine managed rhinology cases effectively.	[18,19,38,69,70,71]
Plastic	Operating and outpatient capacities significantly reduced with adapted anesthesia and shorter procedures, though telemedicine enabled ongoing patient management and training despite limitations.	[24]
Pediatric	Procedures requiring anesthesia down 55% with 40% fewer pediatric fractures but longer follow-up times, while telemedicine use increased 75% though equitable access requires further investigation.	[30,61,72,73,74]

**Table 4:** Changes in surgical practices during COVID-19 as perceived by surgeons.

Category	Key Findings	Reference
Surgeon availability	Surgeon absences unlikely to limit elective surgery capacity significantly.	[75,76]
Surgical volumes	Widespread reductions in elective, cancer, emergency surgeries.	[77]
Workforce deployment	Reallocation of surgical staff to other hospital services.	[77]
COVID-19 care	Most surgeons continued urgent surgeries on infected patients.	[77]
Pandemic management	Need for communication, leadership, planning, training, regulation.	[79]
Telehealth use	About 85% of surgeons used telehealth during pandemic.	[79]
Telehealth perceptions	Only 38% viewed telehealth as equivalent to in-person consults.	[79]
Telehealth limitations	Issues with physical exam, serious news, conflicts.	[79]
Telehealth barriers	Medicolegal, technical, financial barriers key to address.	[80]

**Table 5:** Hospital policy responses to the COVID-19 pandemic: a cross-study synthesis.

Category	Key Findings	References
Elective surgery	Widespread halting of non-urgent elective procedures.	[31,79]
Workforce policies	Redeployment of residents; flexibility in duty hours.	[31]
Infection control	Enhanced staff training; specialized COVID-19 wards.	[48,82]
Digital health	Expansion to improve access amidst disruptions.	[81]
Telehealth	Over 85% of surgeons used virtual consults; desire continued access post-pandemic.	[79]
Guideline updates	Continuous revision of policies as new evidence emerges.	[82]
Risk assessed-MeNTS	Structured prioritization to guide case reductions.	[9]
Backlog management	Planning needed to minimize adverse outcomes from delays.	[82]

## Data Availability

Data are contained within the article.

## Supplementary Materials

The following supporting information can be downloaded at: https://www.mdpi.com/article/10.3390/healthcare12010096/s1, Table S1. Characteristics of included studies.
