# Supplementary material for: Optimizing Surgical Care Amidst COVID-19: A Scoping Review of Practices and Policies
Source: Healthcare (Basel). 2023 Dec 31;12(1):96. doi: 10.3390/healthcare12010096 (PMC10779415; doi:10.3390/healthcare12010096)
Supplement: Supplementary file 1 [file healthcare-12-00096-s001.zip › healthcare-2749502-supplementary.pdf]

Table S1. Characteristics of included studies.

S.No	Author(s)	Sample	Findings
1	Francis, N., et al.	N/A	Guidelines for safe minimally invasive surgery during COVID-19, emphasizing protective measures.
2	Frakes, M.A., et al.	N/A	Study on adapting critical care transport during the pandemic with a focus on new protocols and adaptability.
3	Prachand, V.N., et al.	N/A	Introduction of a scoring system for prioritizing time-sensitive surgeries during COVID-19.
4	ACS	N/A	Recommendations for triaging non-emergency surgeries during the pandemic.
5	Ksenak, C.	N/A	Statement on safely resuming hospital and office-based procedures amid COVID-19.
6	Saleeby, E., et al.	392 female gynecology patients	Evaluation of a modified surgical prioritization tool in gynecology during the pandemic.
7	Teklewold, B., et al.	N/A	Reduced preoperative waiting times for elective surgeries in Ethiopia during the pandemic.
8	Wee, L.E., et al.	N/A	Emphasizing early COVID-19 detection in surgical patients.
9	Wake, R.M., et al.	N/A	Triage system to reduce in-hospital COVID-19 transmission.
10	Hsu, C.H., et al.	N/A	Triage protocol for post-surgery patients to prevent COVID-19 spread.
11	Stone, R. and S. Scheib	Not provided	Benefits of enhanced recovery protocols in perioperative care during the pandemic.
12	Ljungqvist, O., et al.	Not provided	Discussing improved-expanded form of MeNTS and challenges in enhanced recovery after surgery.
13	Matava, C., et al.	247 patients	Impact of a weekend surgery program in reducing pediatric surgery backlog in Canada.
14	Wu, J., et al.	Not provided	Mobilization of resources for full surgical service capacity in Wuhan.
15	Bose, S.K., et al.	Cases abstracted from the Nationwide Inpatient Sample (2016-2017)	Estimating the financial impact of canceled elective surgeries nationally.
16	Dobbs, T.D., et al.	Adult patients undergoing surgery between Jan 1 and Dec 31, 2020 in England and Wales	33.6% reduction in surgical activity in 2020, with over 1.5 million cancelled operations.
17	Gomez, D., et al.	Surgical procedures in Ontario, Canada during COVID-19	Decreased rate of surgical procedures during COVID-19 due to reduction in elective procedures.
18	Kuehner, G., et al.	Kaiser Permanente Northern California members referred to surgical services from Jan 1, 2019 to Jun 13, 2020	Significant shift to telemedicine for preoperative and postoperative encounters during COVID-19.

19	Schulberg, S.P., et al.	COVID-19 patients in a New York City hospital	Surgical residents team improved efficiency of care for critical COVID-19 patients.
20	Baoas, S.D., et al.	Confirmed COVID-19 cases in Brooklyn, New York from March-May 2020	Presence of surgery had a significant impact on COVID-19 patients discharge destinations.
21	Mattingly, A.S., et al.	Pediatric and adult patients undergoing surgical procedures in 49 US states	Initial shutdown associated with decrease in surgical procedure volume to nearly half of baseline rates.
22	Low, T.Y., et al.	Not specified	Restructuring the surgical service during COVID-19 in Singapore is feasible and safe.
23	Bugaev, N., et al.	Patients treated by an ACS service between January and May 2020 in an urban tertiary academic medical center	Trauma and emergency general surgery volumes decreased during the COVID surge.
24	Chu, K.M., et al.	Six government hospitals in Western Cape, SA	Total general surgery operations decreased by 44% during the COVID-19 lockdown.
25	McLean, R.C., et al.	Emergency general surgery admissions in the UK, before and after lockdown	Following lockdown, a significant reduction in median daily admissions.
26	Welk, B. and L. Richard	Individuals with a positive COVID-19 test and their matched controls in Ontario, Canada	Following COVID-19, there is not an increased risk of needing several common surgical procedures.
27	Guadalajara, H., et al.	ASIP cases from 2019, 2020 and 2021 (March 14th to May 2nd) in Spain	The number of patients treated for ASIP reduced by 1/3rd during the first COVID -19 wave.
28	Rovers, M.M., et al.	A large hospital in the Netherlands	Development of a surgical prioritisation framework showing the highest loss in quality of life due to delayed surgery.
29	Sa, A.F., et al.	Patients who underwent urgent/emergency surgery between March 1st and May 2nd of both 2020 and 2019 in Portugal	30% less patients underwent urgent/emergency surgery during the COVID-19 pandemic.
30	Laas, D.J., et al.	Tertiary hospital in KwaZulu-Natal Province, South Africa	Theatre caseload decreased by 30% from January to April 2020.
31	Pardolesi, A., et al.	83 patients with lung cancer	Telemedicine program for lung cancer patients was effective and preferred.

32	Hinchcliffe, Z., et al.	327 patients undergoing day case laparoscopic cholecystectomy (expanded form of DCLC)	Identifying patient factors linked to successful expanded form of DCLC in isolated day-case units during the pandemic.
33	Passoni, R., et al.	Brazilian National Transplant System data analysis	Kidney transplant activities in Brazil decreased by 23.9% during the pandemic.
34	Sukmanee, J., et al.	Universal Coverage Scheme data for acute appendicitis patients from 2016 to 2021	Reduction in acute appendicitis cases during lockdown in Thailand suggests potential overdiagnosis.
35	Teoh, J.Y., et al.	1004 urological service providers	Global impact of COVID-19 on urological care and providers.
36	Lambracos, S., et al.	Urology service data during NHRFA level 2	Significant decrease in outpatient consultations and procedural clinic appointed-expanded form of MeNTS in urology.
37	Hussain, A., et al.	2600 cardiac surgery patients	Unexpected reduction in sternal wound infections during the COVID-19 pandemic.
38	Vlastos, D., et al.	Patients operated on from March 2020 to May 2020	Impact of the pandemic on aortic valve surgical service, with a 70% reduction in elective cases.
39	McPherson, I., et al.	Patients receiving open and endovascular thoracic aortovascular intervention	Increase in urgent cases of thoracic aortovascular disease in 2020, with no significant outcome differences.
40	Leung, S., et al.	N/A	Study on the evolving impact of COVID-19 on surgical services, with no specific findings provided.
41	Al-Thani, H., et al.	All vascular outpatient encounters during 2019 and 2020	61% of total patient encounters in 2020 were via teleconsultation in vascular surgery.
42	Chen, A.J., et al.	94 patients and 144 telemedicine visits over a 22-month period	Telemedicine provided safe, efficient care during the pandemic and saved travel for patients.
43	Traina, L., et al.	PAD patients requiring revascularization between March 2019 and March 2021	More severe stages of limb ischemia during the pandemic periods.
44	Veraldi, G.F., et al.	Vascular procedures performed between March 2019 and December 2019 (prepandemic) compared to March–December 2020 (pandemic).	Increase in limb-threatening ischemia and major limb amputation during the pandemic.

45	Sutjiadi, B., et al.	Referrals to oncologic surgical specialty clinics at an academic tertiary care institution following implementation of stay-at-home orders in California	Decrease in patients seen in oncologic surgical specialty clinics during COVID-19, with higher acuity cases.
46	Specht, M., et al.	Consecutive patients undergoing immediate breast reconstruction	Successful same-day breast reconstruction during the COVID-19 crisis with no complications.
47	Waters, R., et al.	Orthopaedic surgical cases in South Africa	Drastic reduction in orthopaedic services during COVID-19 in South Africa.
48	Avant-Garde, H., et al.	All Medicare fee-for-service beneficiaries undergoing shoulder arthroplasty	Decrease in shoulder arthroplasty volume during the pandemic, but shorter hospital stays.
49	Sharkey, S., et al.	Data on theatre timings and procedures	Significant reduction in theatre efficiency due to COVID-19 restrictions in the UK.
50	Karanjia, R., et al.	Patients undergoing orthopaedic trauma surgery in the UK	Increased delays and overall case time in orthopaedic trauma surgery during COVID-19.
51	Kulkarni, K., et al.	Orthopaedic trauma care at a university teaching hospital	Paradigm shift in trauma care due to COVID-19, leading to more cost-effective practices.
52	Jeyaseelan, L., et al.	Theatre productivity analysis in London	Modest delays in theatre use and decreased efficiency due to COVID-19 in major trauma centres in the UK.
53	Hall, A.J., et al.	Survey of senior clinicians or service managers	Disruption to hip-fracture services during COVID-19, affecting surgical facilities and theatre efficiency.
54	Leti Acciaro, A., et al.	Retrospective study in hand surgery and microsurgery	Significant reduction in surgical procedures in hand surgery and microsurgery during COVID-19.
55	Chan, Y., et al.	Compilation of best evidence on rhinologic and skull-based surgeries	Guidelines for safely restarting rhinologic and skull-based surgeries during COVID-19.
56	Hsieh, T.Y., et al.	Guidelines for facial trauma procedures	Stratification and protective measures for facial trauma procedures during COVID-19.
57	George, C.K., et al.	Experience of emergency tracheostomy in a surgical oncology unit	Challenges of performing emergency tracheostomy during COVID-19 due to aerosolization risk.
58	Krishnamoorthy, S., et al.	143 ventilator-dependent COVID-19 patients undergoing tracheostomy	Safe and efficient tracheostomy in ventilator-dependent COVID-19 patients.

59	Sethia, R., et al.	Head and neck surgery patients undergoing at-home drain removal	Safe and efficacious at-home drain removal in head and neck surgery patients.
60	Alshareef, M., et al.	Adult rhinology cases receiving telemedicine care	Effective use of telemedicine in managing rhinology cases during the COVID-19 pandemic.
61	Johal, K.S., et al.	Pediatric fracture patients in an urban pediatric emergency department	Decline in pediatric fractures during COVID-19, with longer times to surgical follow-up.
62	Lapsa, J., et al.	N/A	Impact of COVID-19 on pediatric fracture patterns and follow-up care in an urban emergency department.
63	Sullivan, J.E., et al.	In-hospital pediatric surgery consultations during early COVID-19	More efficient triaging for pediatric surgical consultations in the emergency department during early COVID-19.
64	Mallenahalli, S., et al.	377 pediatric urologists globally	Significant disruptions in pediatric urology services due to COVID-19, with increased telemedicine use.
65	Cockrell, H.C., et al.	Pediatric patients seen by surgical or pre-anesthesia providers	Telehealth use for pediatric surgery reduced patient travel and CO2 emissions, but had lower use among minorities.
66	Shrestha, B.M.	Not specified	Discussion on delivering surgical services during the COVID-19 pandemic.
67	Collaborative, C.O.	Not specified	Study on surgical workforce availability during COVID-19, predicting sufficient staffing for elective surgeries.
68	Chu, K.M., et al.	Surgeons working in South African hospitals	Changes in surgical practice in South Africa during COVID-19 lockdown, including reduced operations.
69	Patriti, A., et al.	Surgeons from multiple Italian regions	Emergency general surgery reorganization in Italy during COVID-19, with varied compliance with recommendations.
70	Alanezi, F., et al.	336 healthcare practitioners in Saudi Arabia	Positive attitudes among Saudi healthcare practitioners towards managing COVID-19 outbreak.
71	Wiadji, E., et al.	683 Australian Fellows of the Royal Australasian College of Surgeons	Surgeons in Australia adopted telehealth during the pandemic, noting its limitations and benefits.
72	Edwards, J.A., et al.	Not specified	Surgical training restructuring and maintaining surgical services during COVID-19.
73	Alhodaib, H. and T.M. Alanzi	1698 Saudi Arabian citizens	Increased adoption of digital health technologies in Saudi Arabia during COVID-19.
74	Ma, X., et al.	Not specified	Impact of COVID-19 on emergency and essential surgical healthcare in low- and middle-income countries.
75	Weber LeBrun, E.E., et al.	Not specified	Adjusting surgical services for gynecology and obstetrics during the COVID-19 pandemic.
